# Radiologic Predictors of Disease Recurrence in Nasopharyngeal Carcinoma: A Retrospective Evaluation of MRI and ^18^F-FDG-PET/CT Parameters

**DOI:** 10.3390/diagnostics15131646

**Published:** 2025-06-27

**Authors:** Banu Karaalioğlu, Tansel Çakır, Ömer Yazıcı, Mustafa S. Tekin, Ebru Karcı

**Affiliations:** Departments of Radiology, Nuclear Medicine, Radiation Oncology, Otorhinolaryngology and Medical Oncology, Istanbul Medipol University Hospital, Istanbul 34214, Turkey; drtansel@gmail.com (T.Ç.); yzcomer@yahoo.com (Ö.Y.); drmustafasaidtekin@gmail.com (M.S.T.); dr.ebrukarc@yahoo.com (E.K.)

**Keywords:** nasopharyngeal carcinoma, MRI, ^18^F-FDG-PET/CT, imaging predictors of disease recurrence, MRI response assessment

## Abstract

**Background/Objectives:** NPC is a radiosensitive malignancy with high recurrence rates despite therapeutic advances. This study aimed to identify radiologic and metabolic predictors of recurrence in newly diagnosed NPC by integrating MRI and ^18^F-FDG PET/CT parameters. **Methods:** Fifty-two patients with biopsy-proven, previously untreated NPC who underwent pre-treatment MRI and ^18^F-FDG PET/CT were retrospectively analyzed. Local tumor features, nodal status, and response patterns were evaluated using MRI and PET/CT-derived metrics: SUVmax, SUVmean, SUVpeak, MTV, and TLG. The post-treatment MRI response was categorized into six patterns. Univariate and multivariate analyses were performed to identify independent predictors. **Results:** Recurrence occurred in 27% of patients. Based on the multivariate analysis, PNI, extensive PPS invasion, GTV, and metastatic LN count were identified as independent predictors of recurrence (PNI: OR = 1.60, *p* = 0.029; PPS: OR = 1.23, *p* = 0.027; GTV: OR = 1.08, *p* = 0.042; LN count: OR = 1.20, *p* = 0.031). PNI and PPS invasion were significantly associated with local failure (HR = 8.21, *p* = 0.008 and HR = 3.52, *p* = 0.043, respectively). GTV was independently associated with an increased risk of local (HR = 1.14, *p* = 0.021) and distant recurrence (HR = 1.19, *p* = 0.024). The presence of metastatic disease at diagnosis (HR = 6.27, *p* = 0.027) and a higher LN count (HR = 1.17, *p* = 0.028) were also linked to distant failure. **Conclusions:** Imaging-derived predictors including GTV, PNI, LN burden, and MRI-based response patterns demonstrate prognostic value for disease recurrence in NPC and may guide risk-adapted treatment strategies.

## 1. Introduction

Nasopharyngeal carcinoma (NPC) is a malignant epithelial tumor arising from nasopharyngeal mucosa and accounted for 0.8% of cancer-related deaths worldwide in 2020 [1]. Despite its global rarity, NPC demonstrates a strikingly unbalanced geographic distribution, with more than 70% of new cases reported in East and Southeast Asia [2]. In endemic regions, it comprises approximately 28–29% of all head and neck malignancies [3,4,5]. However, NPC is biologically and clinically distinct from other head and neck epithelial tumors, exhibiting an insidious onset, a high propensity for early metastasis, and a unique association with Epstein–Barr virus (EBV) infection [2].

Due to its deep anatomical location, clinical presentation is often delayed and typically reflects the extent of the primary tumor or more frequently regional lymphatic involvement. Cervical lymphadenopathy is the most common initial finding, observed in approximately 75% of patients [6]. Fewer than 7% of patients present with stage I disease; most are diagnosed at a locoregionally advanced stage, and about 10–15% present with distant metastases at the time of diagnosis [7,8]. NPC’s aggressive and infiltrative growth within a complex anatomical region presents significant therapeutic challenges, often rendering complete surgical resection unfeasible. Its high radiosensitivity has made radiotherapy the cornerstone of treatment. Intensity-modulated radiotherapy (IMRT), with or without concurrent chemotherapy, remains the standard approach [2]. For early-stage disease, IMRT alone is considered sufficient, while concurrent chemoradiotherapy (CCRT) is the preferred strategy for locoregionally advanced disease [7,9].

The survival benefit of adding adjuvant chemotherapy following CCRT remains controversial [10]. In contrast, the addition of induction chemotherapy prior to CCRT has demonstrated improved overall survival, largely due to the better control of distant metastases [11]. In the era of IMRT, both strategies are endorsed in clinical guidelines and continue to play key roles in the management of advanced-stage NPC [12]. Nevertheless, despite these advances, 3–13% present with residual disease within 12 weeks post-treatment and 20–30% of patients still develop local recurrence or distant metastasis [13,14,15].

The 8th edition of the UICC/AJCC TNM staging system remains the most widely adopted and universally accepted standard for prognostic stratification in NPC [16]. MRI, CT, and ^18^F-FDG-PET/CT are the most commonly employed imaging modalities for this purpose. Owing to its significantly superior soft-tissue resolution, MRI is more effective in evaluating primary tumor extension and retropharyngeal lymph node involvement, while ^18^F-FDG-PET/CT is more accurate for detecting distant metastases and identifying small cervical lymph node metastases [17,18,19,20]. Unfortunately, clinical outcomes often vary among patients with the same TNM stage receiving similar treatment, highlighting the need for more precise prognostic markers and individualized therapeutic approaches.

This study investigates risk factors for disease recurrence in NPC by systematically evaluating local tumor characteristics, nodal involvement, and treatment response patterns through the integration of MRI findings with PET/CT-derived metabolic parameters.

## 2. Materials and Methods

Medical records of 80 consecutive patients diagnosed with NPC between January 2018 and February 2022 at our institution were retrospectively reviewed. All patients had biopsy-confirmed, previously untreated disease. Patients with stage I disease (n = 5), those who lack follow-up (n = 12), or those with incomplete baseline imaging (n = 11) were excluded from this study. The final study cohort comprised 52 patients who had undergone comprehensive baseline imaging—including concurrent contrast-enhanced neck MRI and ^18^F-FDG-PET/CT—and maintained regular clinical and radiological follow-up for at least 3 years.

All patients were restaged according to the 8th edition of the AJCC staging system. Pre-treatment primary tumor volume, laterality of the tumor (unilateral or bilateral), and growth pattern (mass-forming vs. infiltrative) were documented. Tumor volume was manually contoured on each axial slice of pre-treatment contrast-enhanced T1-weighted MRI using the syngo.via^®^ VB60 image analysis software (Siemens Healthineers, Erlangen, Germany). Segmentations were performed by a single radiologist with nine years of experience in head and neck imaging. After manual delineation, gross tumor volume (GTV) was automatically calculated by the syngo.via^®^ VB60 software based on the contoured areas and the predefined slice reconstruction interval of 3 mm.

**Figure 1:**
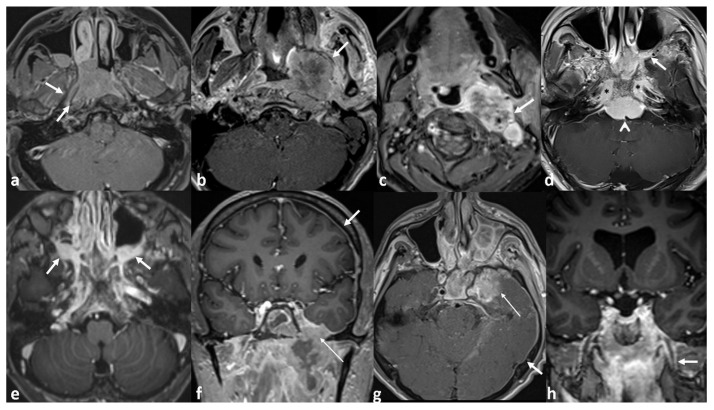
Post-contrast axial and coronal T1-weighted MR images showing patterns of tumor extension. (**a**) Tumor extends beyond the pharyngobasillary fascia but is confined to the tensor veli palatini. (**b**) Mass-forming left-sided tumor invading the lateral pterygoid muscle (arrow), indicating extended PPS invasion. (**c**) Extended PPS invasion with ICA encasement in the carotid space (arrow). (**d**) ICS and skull base invasion involving the foramen lacerum (stars), left pterygopalatine fossa (arrow), and prepontine cistern (arrowhead). (**e**) Infiltrative pattern with bilateral enlargement of pterygopalatine fossae. (**f**) CN V involvement with widening of the foramen ovale (thin arrow) and dural infiltration (thick arrow). (**g**) Parenchymal invasion of the medial temporal lobe, dural spread (arrows), and cavernous ICA invasion. (**h**) Perineural spread along CN V (arrow). CN, cranial nerve; ICA, internal carotid artery; PPS, parapharyngeal space; SB, skull base; ICS, intracranial space.

Tumor extension was evaluated in detail beyond AJCC staging; parapharyngeal space (PPS), skull base (SBI), and intracranial space (ICS) invasions were further categorized as ‘local’ or ‘extended’ based on the depth of anatomical involvement. Classification criteria are summarized in Table 1. Perineural invasion (PNI) was included as an independent predictor of disease recurrence. Figure 1 presents representative MRI examples illustrating various patterns of local tumor extension.

Lymph node metastases identified by ^18^F-FDG PET/CT were further assessed from concurrent MRI in addition to the AJCC nodal classification, including nodal dimensions (short and long axis), the presence of necrosis, and the number of metastatic lymph nodes. Nodal positivity was confirmed by complete resolution on follow-up imaging after chemoradiotherapy.

**Figure 2:**
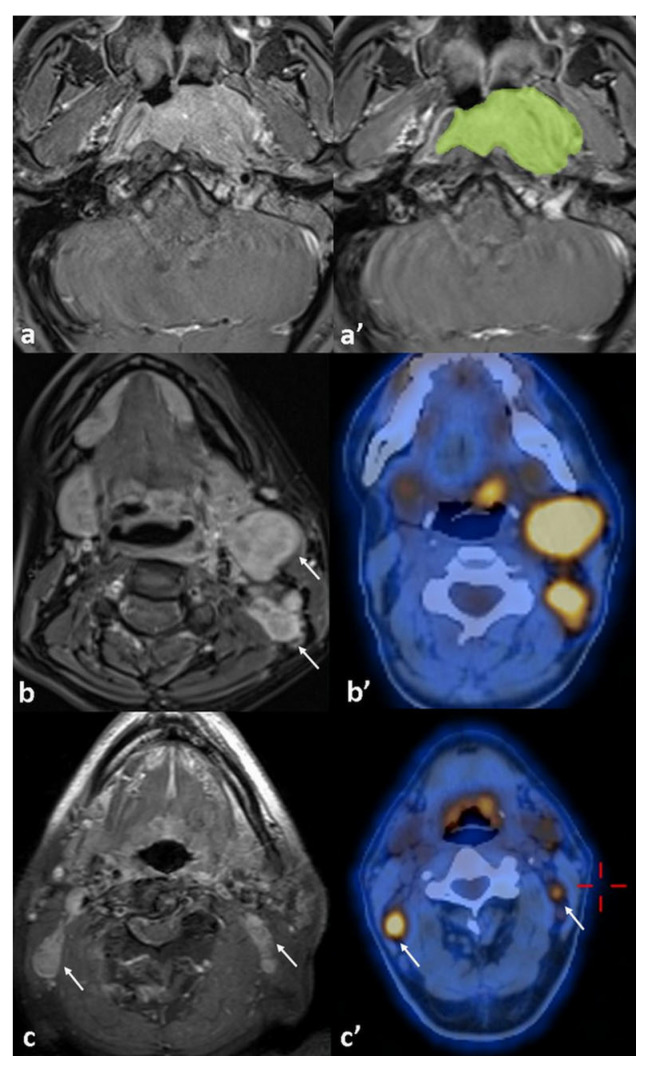
(**a**) Axial contrast-enhanced T1-weighted MR image shows the primary tumor located on the left lateral wall of the nasopharynx. (**a**′) Gross tumor volume (GTV) measurement is demonstrated on the same axial slice using the image viewer in syngo.via^®^ VB60 (Siemens Healthineers). The tumor was manually delineated on each axial slice where it was visible, and the software automatically calculated the total volume by summing the segmented areas across slices. The segmented tumor regions were automatically color-coded by the software (green area). (**b**,**b**’) Metastatic lymph nodes at left cervical levels IIb and Va are visualized on concurrent MRI and fused ^18^F-FDG PET/CT images (arrows). (**c**,**c**’) Bilateral small but pathological lymph nodes are demonstrated on both MRI and fused PET/CT images (arrows).

Baseline metabolic parameters derived from ^18^F-FDG-PET/CT—including peak standardized uptake value (SUV-peak), maximum SUV (SUV-max), mean SUV (SUV-mean), metabolic tumor volume (MTV), and total lesion glycolysis (TLG)—were measured for both the primary tumor (PT) and the most metabolically active metastatic lymph node (LN) to assess their association with disease recurrence. Figure 2 displays an example of tumor volume measurement, accompanied by imaging of lymph node involvement on ^18^F-FDG PET/CT and MRI.

All patients were treated according to a standardized institutional protocol, consistent with current international guidelines. All patients received CCRT with cisplatin and IMRT. Chemotherapy regimens were selected based on tumor stage and nodal burden, as determined during multidisciplinary tumor board discussions. Patients receiving induction chemotherapy were treated with cisplatin and gemcitabine, while those undergoing maintenance therapy received oral capecitabine. These treatment decisions were made according to a consistent institutional algorithm. Additionally, to account for potential treatment-related variability, clinical stage was included as a covariate in multivariate models. This approach helped reduce confounding and strengthened the internal validity of our findings.

Follow-up MRI and ^18^F-FDG-PET/CT scans were reviewed for all patients in the cohort to assess residual disease or recurrence. The optimal radiologic response to treatment was determined based on follow-up MRI findings. MRI characteristics at the time of best response were evaluated across T1WI, T2WI, STIR, ADC maps, and post-contrast T1WI sequences. Radiologic response patterns were classified into six categories: (1) complete tumor resolution (absence of any residual tumor or sequel signal abnormality), (2) thin fibrosis at the primary tumor site, (3) bulky fibrosis without discernible soft tissue or enhancement, (4) heterogeneous signal with subtle contrast enhancement, (5) residual enhancing soft tissue, and (6) complete response at the primary site with persistent nodal disease. Associations between response patterns and local tumor stage as well as local failure were subsequently analyzed.

All statistical analyses were performed using Stata version 18.0 (StataCorp LLC, College Station, TX, USA). Tumor characteristics derived from MRI and ^18^F-FDG-PET/CT metabolic parameters were compared to assess their association with disease recurrence. Chi-square tests were applied for categorical variables, and independent t-tests were used for continuous variables, as appropriate. Variables demonstrating statistical significance in univariate analyses (*p* < 0.05) were subsequently included in a multivariate logistic regression model to determine their independent predictive value for recurrence. The Chi-square test was conducted to evaluate the association between MRI treatment response patterns and recurrence risk. To assess the strength of association between post-treatment MRI response patterns and initial T-stage, Kendall’s Tau-b correlation was used. In addition, variables with *p* < 0.05 in univariable analysis were included in a multivariable Cox regression model to assess their independent association with time-to-recurrence outcomes. Hazard ratios (HRs) with 95% confidence intervals (CIs) were reported. A two-sided *p* value < 0.05 was considered statistically significant.

MR imaging was performed on 1.5-Tesla machine (Sola; Siemens). Routine sequences arranged for all neck imaging in our institution were used for the patients’ MRI scans. The sequences were acquired with a 20-array head coil and included the following data; axial and coronal T2-TSE-Dixon (3450/85; TR/TE, slice thickness 3 mm, matrix: 320 × 224; 4380/83; TR/TE, slice thickness 3 mm, matrix: 320 × 232), axial DWI-b1000 (4500/67; TR/TE, slice thickness 3.5 mm, matrix: 128 × 128) and pre-contrast axial and post-contrast 3 plan T1-TSE-Dixon (413/13; TR/TE, slice thickness 3 mm, matrix: 198 × 288; 440/13; TR/TE, slice thickness 3 mm, matrix: 217 × 320; 524/13; TR/TE, slice thickness 3 mm, matrix: 210 × 320).

PET/CT imaging was performed using a Siemens Biograph mCT scanner. Patients received an intravenous injection of approximately 370 MBq (10 mCi) of 18F-FDG and rested for 60 min before scanning. PET data were acquired in 3D mode over 7 bed positions, 3 min per position, and reconstructed using a 200 × 200 matrix with point spread function (PSF) and time-of-flight (TOF) modeling (3 iterations, 21 subsets). Low-dose CT (120 kV, ~57 mAs) was performed for attenuation correction and anatomical localization. Images were corrected for attenuation, scatter, and random events, and reviewed on a dedicated workstation.

## 3. Results

A total of 52 patients with newly diagnosed NPC were included in this study. The cohort comprised forty-three males and nine females, with a mean age of 46 ± 12 years (range: 20–69). All tumors were of the nonkeratinizing subtype, with 31 classified as differentiated and 21 as undifferentiated. According to the 8th edition of the AJCC staging system, 25% (thirteen out of fifty-two) were diagnosed with stage II, 29% (fifteen out of fifty-two) with stage III, 31% (16/52) with stage IVA, and 15% (eight out of fifty-two) with stage IVB disease.

Among the eight patients with metastatic disease, seven had bone involvement—two with isolated vertebral lesions and five with additional metastases including distant lymph nodes (n = two), liver (n = two), and lung (n = one). One patient had isolated lung metastasis.

Disease recurrence was observed in 14 patients (27%) and confirmed through biopsy or serial imaging. Three additional patients—two with skull base osteitis secondary to sphenoid sinusitis on MRI and one with mucositis demonstrating FDG avidity on PET/CT—were initially suspected of recurrence but were subsequently confirmed as false positives. Among the fourteen confirmed recurrences, one patient had residual enhancing soft tissue on MRI at 4 months post-treatment, which remained stable before progressing locally at 8 months. The remaining thirteen patients developed recurrence following an initial complete remission confirmed by both MRI and PET/CT: eight had local failure, five had distant failure, and one had both local and distant failure. Among the six patients with distant metastases, four (66.7%) had metastatic disease at initial diagnosis. Secondary relapse occurred in 57% (eight out of fourteen) of recurrent cases, and five of the them relapsed after a complete response. Demographic and tumor-specific characteristics are summarized in Table 2.

**Figure 3:**
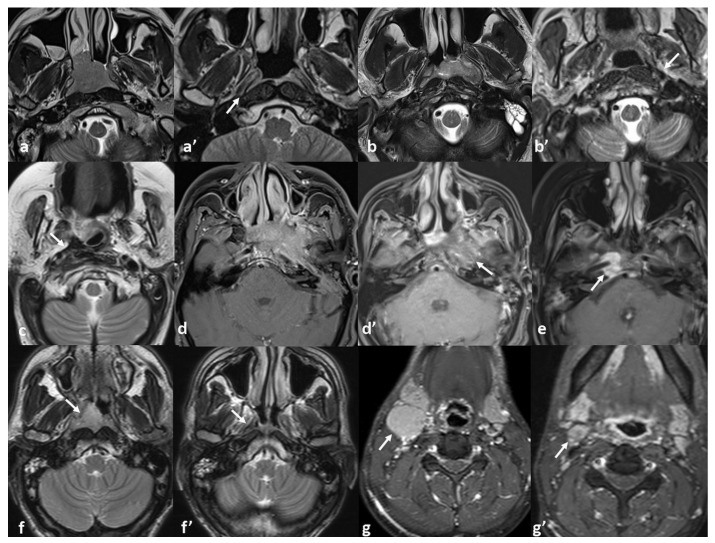
Pre- and post-treatment MRI findings demonstrating response patterns. Original letters, e.g., (**a**), indicate pre-treatment images; apostrophes, e.g., (**a**’), indicate corresponding post-treatment images. Axial TSE-Dixon T2WIs (**a**–**c**,**f**) and post-contrast fat-saturated axial TSE-Dixon T1WIs (**d**,**e**,**g**) are shown. (**a**,**a**’) Complete response with no residual or post-treatment changes. (**b**,**b**’) Linear fibrotic changes at the original tumor site (arrow). (**c**) Bulky fibrosis obliterating fat planes, without intermediate signal or soft tissue component (arrow). (**d**,**d**’) Heterogeneous signal with subtle enhancement, without soft tissue residue (arrow). (**e**) Residual enhancing soft tissue at the foramen lacerum (arrow). (**f**,**f**’) Complete local response (arrow) with (**g**,**g**’) residual level IIa lymph node (arrow).

Seventeen patients demonstrated completely normal post-treatment MRI findings, with no evidence of residual disease or treatment-related sequelae. However, ten patients (19%) showed thin localized fibrosis, eleven (21%) had bulky non-enhancing fibrosis, ten (19%) exhibited heterogeneous signal with subtle enhancement, and four (8%) presented with residual enhancing soft tissue at the primary tumor site. Post-treatment MRI response patterns showed a moderate to strong correlation with the initial T-stage (Kendall’s tau-b = 0.43, *p* < 0.001). Advanced-stage tumors more frequently exhibited category 3–5 response patterns—bulky fibrosis, heterogeneous enhancement, or residual enhancing tissue—which were also significantly associated with disease recurrence (*p* = 0.004). Post-treatment MRI response patterns and the association with local tumor stage and recurrence status are detailed in Table 3. Figure 3 presents MRI findings demonstrating diverse patterns of tumor response.

The univariate analysis showed that the overall disease stage is a significant predictor of recurrence, with recurrence rates increasing across advancing stages (*p* = 0.044). Patients with metastatic disease had significantly higher recurrence than non-metastatic patients (62.5% vs. 20.4%, *p* = 0.014). Tumors with bilateral mucosal involvement had higher recurrence rates compared to unilateral cases (54.5% vs. 22.8%, *p* = 0.034), and infiltrative growth patterns were more likely to recur than well-defined, mass-forming tumors (62.5% vs. 20.4%, *p* = 0.014). Primary tumor stage (T-stage) did not reach statistical significance (*p* = 0.06); however, a detailed local analysis revealed that PNI was a strong predictor of recurrence (77.7% vs. 16.2%, *p* < 0.001). Extensive PPS invasion (62.5% vs. 11%, *p* = 0.001) and the presence of ICS invasion (63.6% vs. %17, *p* = 0.008) were also significantly associated with recurrence, whereas SBI was not (35% vs. %21.8, *p* = 0.109).

Among nodal parameters, only the number of metastatic lymph nodes was significantly associated with disease recurrence (*p* = 0.020). Recurrence was also more frequent in patients with necrotic nodes compared to those without (47% vs. 17.1%, *p* = 0.023), whereas the nodal stage and lymph node diameter showed no significant association.

Among PET/CT-derived metabolic parameters, only MTV (*p* = 0.049) and TLG (*p* = 0.045) demonstrated marginal significance for disease progression in the univariate analysis. However, none retained independent prognostic value in the multivariate model.

The multivariate analysis identified PNI, extensive PPS invasion, tumor volume, and the number of metastatic lymph nodes as independent predictors of recurrence. PNI was associated with a 60% increase in the odds of recurrence (OR = 1.60, *p* = 0.029), while extensive PPS invasion conferred 23% higher odds (OR = 1.23, *p* = 0.027). Each cm^3^ increase in tumor volume was linked to an 8% increase in the odds of recurrence (OR = 1.08, *p* = 0.042), and each additional metastatic lymph node raised the odds by 20% (OR = 1.20, *p* = 0.031). Results of the univariate and multivariate analyses are summarized in Table 4.

Both PNI and extensive PPS invasion were significantly associated with local recurrence (PNI: HR = 8.21; 95% CI: 3.73–28.37; *p* = 0.008; PPS: HR = 3.52; 95% CI: 1.39–11.9; *p* = 0.043). Tumor volume emerged as a common independent predictor linked to both local and distant recurrence (local: HR = 1.14; 95% CI: 1.02–1.29; *p* = 0.021; distant: HR = 1.19; 95% CI: 1.05–1.40; *p* = 0.024). The presence of metastatic disease at diagnosis (HR = 6.27; 95% CI: 1.23–31.9; *p* = 0.027) and a higher number of metastatic lymph nodes (HR = 1.17; 95% CI: 1.08–1.35; *p* = 0.028) were associated with increased risk of distant failure.

Local recurrences were managed with CyberKnife radiosurgery, while distant metastases were treated with systemic chemotherapy. By the end of follow-up, seven patients (13%) had died from cancer-related causes, forty-three were alive, and two were lost to follow-up after three years.

## 4. Discussion

This study identified PNI, extensive PPS invasion, tumor volume, and the number of metastatic lymph nodes as significant predictors of disease recurrence in NPC. PNI and extensive PPS invasion emerged as independent predictors of recurrence and were significantly associated with an increased risk of local failure. Prior studies have also highlighted the adverse prognostic implications of spatial tumor spread. Liu X. et al. reported MRI-detected cranial nerve invasion as an independent predictor of local recurrence (HR = 2.605, *p* = 0.032), consistent with our findings regarding PNI [21]. Although carotid space invasion was not directly assessed in our cohort, it is classified under extensive PPS involvement and has been identified as a high-risk feature in previous studies [22]. For example, Huang et al. proposed subclassifying PPS invasion based on its extent to reduce heterogeneity within the T3 category, citing its association with aggressive tumor behavior [23]. Similarly, Zhang et al. demonstrated that invasion beyond the lateral pterygoid muscle was associated with an increased risk of distant failure (HR = 1.734, *p* = 0.006) [24]. These findings highlight the clinical importance of the imaging-based subclassification of spatial tumor extension and the identification of PNI to facilitate early risk stratification and guide treatment planning.

The absence of a significant association between skull base invasion with recurrence may seem inconsistent with the prior literature. However, this discrepancy likely stems from an ambiguous categorization of bone invasion in previous papers. In our analysis, skull base invasion was strictly defined as bony infiltration, whereas PNI was evaluated independently and reported as a separate variable. Cheng et al. reported a significant prognostic impact of skull base invasion in an advanced subgroup that included neural foraminal involvement [25]. Similarly, Feng et al.’s study identified extended skull base invasion as an independent predictor of recurrence; however, as in Cheng et al.’s work, extended invasion included neural foramina [26]. Including neural foraminal invasion as part of extended skull base invasion may lead to misattributing a poor prognosis to bone involvement, when the adverse outcomes may, in fact, reflect the contribution of perineural spread.

Tumor volume was found to be a significant independent predictor of recurrence in the multivariate model (*p* = 0.042). This finding is consistent with previous studies that underscore the prognostic relevance of tumor burden in NPC. Weiqiong et al. reported that GTV was the only independent predictor of local control (HR = 5.24; 95% CI: 1.074–25.57; *p* = 0.041) [27]. Tian et al. similarly demonstrated that patients with GTV > 38 cm^3^ had a significantly increased risk of local failure compared to those with smaller volumes (*p* < 0.01), advocating for corresponding revisions to T-category classification [28]. Although Qin et al. primarily focused on survival endpoints, they also observed poorer outcomes in patients with GTV > 33 mL, indicating the broader prognostic impact of tumor burden even when stratified by T or clinical stage [29]. Collectively, these findings support incorporating tumor volume into future refinements of the TNM staging system to enhance prognostic stratification. However, a wide range of GTV cut-off values has been reported across studies, likely due to differences in cohort characteristics and imaging methodology [30,31,32,33]. Tumor volume demonstrated predictive value for both local and distant recurrence; however, no definitive cut-off was proposed to avoid overgeneralization from a single-center dataset. These findings support the recommendation to incorporate tumor volume into future TNM staging revisions and underscore the need for meta-analytic studies to establish standardized, evidence-based thresholds.

^18^F-FDG PET/CT enables the non-invasive functional assessment of both the primary tumors (PTs) and metastatic lymph nodes (LN). Elevated standardized uptake values (SUVmax, SUVmean, SUV-peak), along with TLG and MTV, reflect either increased glucose metabolism or cellular density. Guo et al. investigated the relationship between ^18^F-FDG PET metabolic parameters (SUVmax, SUVmean, MTV, and TLG) and the Ki-67 labeling index. Their analysis revealed a statistically significant but weak correlation (r < 0.225) between metabolic activity and proliferative status [34]. Aktan et al. demonstrated the potential predictive value of SUVmax, reporting significantly elevated values in patients with unfavorable outcomes (PT ≥ 13, *p* = 0.017; LN ≥ 9, *p* = 0.013) [35]. In another study, Jin et al. identified SUVmax-N as the only independent prognostic factor associated with distant failure in locoregionally advanced NPC [36]. Although higher MTV and TLG values of the primary tumor were observed in pairwise comparisons, these did not remain statistically significant predictors of recurrence in the multivariate analysis. This suggests that the prognostic value of volumetric metabolic parameters may vary across patient populations and methodological settings, limiting their current utility as universal markers for risk stratification.

The prognostic relevance of various characteristics of metastatic lymph nodes in NPC has been previously investigated. Huang et al. identified maximal nodal diameter as a significant prognostic factor, with nodal size ≥ 4 cm associated with more aggressive disease behavior [37]. Chen et al. reported that the co-existence of bilateral retropharyngeal and cervical lymph node metastases independently predicted disease progression and increased the risk of distant failure (*p* < 0.001) [38]. Jiang et al. demonstrated that the involvement of posterior level V nodes was significantly associated with a higher likelihood of distant spread [39]. In our analysis, necrotic lymph node metastasis emerged as a notable risk factor for disease recurrence, consistent with previous studies that have linked nodal necrosis to unfavorable clinical outcomes [40,41,42]. However, this feature did not remain an independent predictor in the multivariate analysis after adjusting for other significant confounding variables. Given its potential clinical value, we recommend that the presence of nodal necrosis be routinely documented in baseline MRI evaluations.

Among the nodal characteristics evaluated, including N stage, maximum nodal diameter, bilateral involvement, and presence of necrosis, only the number of metastatic lymph nodes remained an independent predictor of recurrence risk (OR = 1.20, *p* = 0.031). Furthermore, each additional metastatic lymph node was significantly associated with an increased risk of distant failure (*p* = 0.028). In line with this, Ma et al. reported that patients with ≥9 positive LNs exhibited significantly worse outcomes compared to those classified as N3 [43]. Consistent with our findings, Zhu et al. emphasized the prognostic significance of the metastatic nodal count, identifying it as an independent and superior predictor of distant failure compared to other nodal characteristics [44]. This highlights the prognostic relevance of metastatic nodal burden in NPC, which may surpass traditional indicators such as nodal size or laterality. Collectively, these findings support the integration of the LN count into the NPC staging framework, as is currently implemented in several gastrointestinal cancers.

In this context, the identification of imaging-based markers becomes increasingly important for early risk stratification and treatment planning. Although extensive PPS invasion and PNI are established markers of locally aggressive disease for which induction or adjuvant chemotherapy may be considered, tumor volume and nodal burden—despite their strong association with recurrence and survival—are not yet incorporated into standard treatment criteria. In our retrospective cohort, these MRI-based features were not used to guide therapy. However, our findings emphasize their clinical relevance and suggest they may warrant inclusion in future risk stratification models to support treatment intensification in high-risk patients.

To date, MRI-based treatment response patterns in NPC have not been systematically characterized. One study by Lin et al. investigated this topic by assessing the relationship between post-radiotherapy fibrosis and recurrence, demonstrating a significantly higher recurrence rate in patients with MRI-detected fibrosis 3–6 months after treatment (*p* = 0.021) [45]. However, their analysis was confined to the presence of fibrosis and did not explore the broader range of post-treatment MRI features or their prognostic implications. In our study, the degree of local tumor invasiveness appeared to influence distinct MRI response patterns. Among patients with advanced-stage disease, features such as bulky fibrosis, heterogeneous signal intensity with subtle enhancement, and residual soft tissue components were observed more frequently and were significantly associated with recurrence risk (*p* = 0.004). Although preliminary, these findings underscore the potential prognostic relevance of MRI-based response assessment in NPC. Prospective validation in larger, multicenter cohorts is needed to confirm these imaging patterns and to determine their clinical utility in identifying patients who may benefit from adjuvant or maintenance chemotherapy.

This study has several limitations that warrant consideration. Its retrospective design may introduce selection bias and limits the ability to establish causal relationships. Conducting the analysis at a single institution may affect the generalizability of the findings across diverse clinical settings and populations. The relatively small sample size may reduce statistical power, particularly in subgroup analyses. Additionally, Epstein–Barr virus (EBV) DNA data were not available, preventing the evaluation of a well-established biomarker that could have enhanced risk stratification. Despite these limitations, the study provides meaningful insights by comprehensively assessing recurrence risk factors in NPC. Unlike many prior studies that examined these variables in isolation, our integrative approach offers a more comprehensive understanding of the interplay between clinical, radiologic, and metabolic parameters.

## 5. Conclusions

This study identified PNI, extensive PPS invasion, tumor volume, and the number of metastatic lymph nodes as significant predictors of disease recurrence in NPC. PNI and extensive PPS invasion were strong indicators of local failure, while tumor volume was independently associated with both local and distant recurrence. The presence of metastatic disease at diagnosis and a higher metastatic nodal burden were also linked to an increased risk of distant failure. Although our findings are consistent with previous studies, they provide a meaningful contribution by specifically addressing the prognostic value of these factors in the Caucasian population. This population-specific validation strengthens the generalizability of prior conclusions and highlights the value of imaging-based stratification across different demographic groups. Importantly, distinct MRI-based treatment response patterns were also associated with recurrence risk, suggesting their potential role in identifying patients who may benefit from adjuvant or maintenance therapy.

## Figures and Tables

**Table 1:** Classification criteria for invasion of the parapharyngeal space, skull base, and intracranial structures.

Classification	Number of Patients (%)	Classification	Number of Patients (%)
Limited PPS invasion Parapharyngeal fat Pre-styloid space Medial pterygoid muscle Retropharygeal space Prevertebral space Nasal cavity Medial pterygoid plate	25 (48%) 2 (3%) 2 (3%) 4 (7%) 8 (15%) 3 (5%) 5 (5%)	Extensive PPS invasion Carotid canal Lateral pterygoid muscle Infratemporal fossa Temporal muscle Lateral pterygoid plate Pterygomaxillary fissure Middle ear Mastoid space Paranasal sinus Orbital cavity Para-oropharyngeal space	13 (25%) 3 (5%) 4 (7%) 2 (5%) 5 (5%) 2 (3%) 1 (2%) 1 (2%) 2 (3%) 1 (2%) 1 (2%)
	27/52		16/52
Limited SB invasion Base of sphenoid bone Clivus Petrous bone apex Sphenoid bone greater wing medial border; bony structure around -Vidian canal -Foramen lacerum	13 (15%) 10 (19%) 2 (3%) 4 (7%)	Extensive SB invasion Pterygopalatine fossa Tegmen tympani Sphenoid bone greater wing and bony structure around -Foramen rotundum -Foramen ovale -Jugular foramen -Hypoglossal canal -Foramen magnum	4 (7%) 1 (2%) 6 (12%)
	13/52		7/52
Limited ICS invasion Cavernous sinus Retro-clival space Dura mater (around middle cranial fossa)	7 (13%) 2 (3%) 4 (7%)	Extensive ICS invasion Orbital cavity through orbital fissure and canal Distant dura mater (exp. lateral to temporal lobe) Brain parenchyma	1 (2%) 3 (5%)
	8/52		3/52

PPS: parapharyngeal space, SB: skull base, ICS: intracranial space; due to multifocal involvement, several patients exhibited invasion of more than one anatomical subsite within the same region. Consequently, the sum of individual subsite counts exceeds the total number of patients classified with extended PPS, SBI, or ICS invasion.

**Table 2:** Demographic and tumor-specific characteristics of the study cohort.

Patients’ Characteristics	No. of Patients (%)
Gender (M/F)	43/9
Age (yrs), mean ± sd	46 ± 12
Pathology (Nonkeratinizing SCC)	
Differentiated	31
Undifferentiated	21
T-Stage (%)	
T1	7/52 (14%)
T2	24/52 (46%)
T3	11/52 (21%)
T4	10/52 (19%)
N-stage	
N0	5/52 (9%)
N1	18/52 (35%)
N2	17/52 (33%)
N3	12/52 (23%)
Overall-Stage (TNM-AJCC 8th addition)	
2	13/52 (25%)
3	15/52 (29%)
4a	16/52 (31%)
4b	8/52 (15%)
Metastasis (n)	8/52 (15%)
Bone	2 (1 = Cervical 5, 1 = Thoracic 6)
Lung	1
Bone + distant LN	2 (n = 1, axilla; n = 1, retroperitoneum)
Bone + Lung	1
Bone + Liver	2
MRI and 18F-FDGPET/CT	
Time interval (d), mean ± sd	3 ± 5
Treatment (n)	
CCRT	28 (54%)
Induction CT + CCRT	10 (19%)
Induction CT + CCRT + maintenance CT	14 (27%)
Disease recurrence	14/52 (27%)
Local	8/14 (57%)
Distant	5/14 (36%)
Both	1/14 (7%)
Second recurrence	8/14 (57%)
Outcome	
Death	7 (13%)
Survive	43 (83%)
Not known	2 (%4)

**Table 3:** Association of post-treatment MRI response patterns with local tumor stage and recurrence status.

MRI Response Patterns	Local Tumor Stage				Total	No Recurrence	Recurrence	Total
	T1	T2	T3	T4				
Complete resolution without any sequel signal abnormality	72% (n = 5)	29% (n = 7)	0% (n = 0)	0% (n = 0)	23% (n = 12)	92% (n = 11)	8% (n = 1)	n = 12
Thin fibrosis at the primary tumor site	14% (n = 1)	21% (n = 5)	36% (n = 4)	0% (n = 0)	19% (n = 10)	90% (n = 9)	10% (n = 1)	n = 10
Bulky fibrosis without discernible soft tissue or enhancement	0% (n = 0)	21% (n = 5)	46% (n = 5)	10% (n = 1)	21% (n = 11)	73% (n = 8)	27% (n = 3)	n = 11
Heterogeneous signal with subtle contrast enhancement	0% (n = 0)	12% (n = 3)	18% (n = 2)	30% (n = 3)	15% (n = 8)	50% (n = 4)	50% (n = 4)	n = 8
Residual enhancing soft tissue	0% (n = 0)	0% (n = 0)	0% (n = 0)	60% (n = 6)	12% (n = 6)	17% (n = 1)	83% (n = 5)	n = 6
Complete resolution at the primary site with persistent nodal disease	14% (n = 1)	17% (n = 4)	0% (n = 0)	0% (n = 0)	10% (n = 5)	100% (n = 5)	0% (n = 0)	n = 5
Total	100% (n = 7)	100% (n = 24)	100% (n = 11)	100% (n = 10)	100% (n = 52)	73% (n = 38)	27% (n = 14)	100% (n = 52)
					*p* < 0.001 * (tau-b = 0.43)			*p* = 0.004 * (χ^2^)

* indicates statistical significance.

**Table 4:** Univariate and multivariate analyses of recurrence risk.

Variables, n (%)		Univariate Analysis *p*	Multivariate Analysis *p*
T-stage		0.060	
T1	1/7 (14%)		
T2	4/24 (16.6%)		
T3	3/11 (27.2%)		
T4	6/10 (60%)		
PT laterality		0.034 *	0.692
Unilateral	8/35 (22.8%)		
Bilateral	6/11 (54.5%)		
Central	0/6 (0%)		
PT Growth Pattern		0.014 *	0.897
Mass-forming	9/44 (20.4%)		
Infiltrative	5/8 (62.5%)		
PPS invasion		0.001 *	0.027 * OR: 1.23 95%CI [1.03–1.49]
No	1/9 (11.1%)		
Local	3/27 (11.1%)		
Extensive	10/16 (62.5%)		
SB invasion		0.521	
No	7/32 (21.8%)		
Local	5/13 (38.4%)		
Extensive	2/7 (28.5%)		
ICS invasion		0.008 *	0.076
No	7/41 (17%)		
Local	5/8 (62.5%)		
Extensive	2/3 (66.6%)		
Perineural invasion		<0.001 *	0.029 * OR: 1.60 95%CI [1.05–2.43]
Positive	7/43 (16.2%)		
Negative	7/9 (77.7%)		
GTV cm^3^ (mean)		0.019 *	0.042 * OR: 1.08 95%CI [1.02–1.16]
No recurrence/recurrence	11.6 (±1.6)/30.9 (±8.2)		
N-stage		0.212	
N0	2/5 (40%)		
N1	2/18 (11.1%)		
N2	7/17 (41.2%)		
N3	3/12 (25%)		
Nodal laterality		0.178	
Unilateral inv.	3/22 (14%)		
Bilateral inv.	9/25 (36%)		
LN diameters (mean ± sd)			
(No recurrence/recurrence)			
Short axis	22.5 (±2)/23.1 (±2.6)	0.432	
Long axis	34.7 (±3.6)/34.4 (±3.9)	0.481	
LN necrosis		0.023 *	0.443
Absent	6/35 (17.1%)		
Present	8/17 (47%)		
No. of Metastatic LN		0.020 *	0.031 * OR: 1.20 95%CI [0.02–0.43]
No recurrence/recurrence	5 (±0.8)/9 (±1.6)		
8th AJCC TNM Stage		0.004 *	
Stage 2	1/13 (7.7%)		
Stage 3	3/15 (20%)		
Stage 4a	5/16 (31.2%)		
Stage 4b	5/8 (62.5%)		
^18^FDG-PET/CT (PT)			0.915 0.757
MTV	21.7 (±3.1)/42.2 (±11)	0.049 *	
TLG	164.4 (±27)/394.2 (±121)	0.045 *	
SUV-max	14.9 (±1.1)/15.9 (±2)	0.328	
SUV-mean	6.8 (±0.4)/7.6 (0.7)	0.195	
SUV-peak	12.2 (±0.9)/13.4 (±2)	0.281	
^18^FDG-PET/CT (LN)			
MTV	19.7 (±4.8)/16.3 (±3.9)	0.295	
TLG	149.4 (±38.8)/139.6 (±42.5)	0.445	
SUV-max	12 (±1.1)/13.5 (±1.9)	0.261	
SUV-mean	5.9 (±0.5)/6.4 (±0.9)	0.314	
SUV-peak	9.4 (±1)/10.9 (±1.8)	0.240	

* indicates statistical significance.

## Data Availability

The data presented in this study are available on request from the corresponding author due to ethical and privacy restrictions.

## Abbreviations

The following abbreviations are used in this manuscript:

NPC	Nasopharyngeal carcinoma
EBV	Epstein–Barr virus
IMRT	Intensity-modulated radiotherapy
CCRT	Concurrent chemoradiotherapy
PPS	Parapharyngeal space
SBI	Skull base invasion
ICS	Intracranial space
PNI	Perineural invasion
SUV	Standardized uptake value
TLG	Total lesion glycolysis
MTV	Metabolic tumor volume
PT	Primary Tumor
LN	Lymph node
DFS	Disease-free survival
LRRFS	Locoregional recurrence-free survival
DMFS	Distant metastasis-free survival
OS	Overall survival
GTV	Gross tumor volume
